# Standardizing DICOM annotation: deep learning enhances body part description in X-ray image retrieval for clinical research

**DOI:** 10.1186/s12880-025-02099-w

**Published:** 2025-12-13

**Authors:** Ka Yung Cheng, Michael Fabel, Björn Bergh, Sylvia Saalfeld

**Affiliations:** 1 Institute for Medical Informatics and Statistics, Kiel University and University Hospital Schleswig-Holstein, Kiel, Germany; 2 Independent Researcher, Kiel, Germany

**Keywords:** Deep learning classification, X-ray image annotation, DICOM, Secondary use, SNOMED CT, Synthetic data, Text-based image retrieval

## Abstract

**Purpose:**

The growing usage of medical imaging for diagnosis and clinical processes provides an increasing amount of materials that can be reused for secondary use. However, this valuable resource often remains underutilized due to non-standardized formatting and annotation. Our study aims to devise a validated annotation model for standardizing and facilitating the reuse of medical images based on real clinical data.

**Methods:**

We extract a dataset with 20k DICOM X-ray images, routinely captured as standard clinical care and stored in the PACS system. A radiologist iteratively annotates and validates 1) examined body parts (single-label pathological classification) and 2) visible body parts (multi-label classification) using 36 relevant SNOMED CT codes.

**Results:**

The proposed model shows an accuracy of 0.889 for classifying examined body parts and 0.853 for classifying visible body parts on the curated dataset. The approach demonstrated advantages in simplicity of use, universal availability, and the ability to enhance data quality. Reducing body parts from 116 distinct DICOM header entries to 36 SNOMED CT codes promises improved retrieval and more concise communication in future applications. In addition, the intersection of Deep Learning models and initial DICOM headers achieved the best result, with a recall of 98.7% in our simulated use case.

**Conclusion:**

Deep learning techniques show potential to address data standardization and quality issues, offering a technically feasible and cost-effective solution for annotating and reusing diverse medical images. Future work should enhance accuracy via multi-radiologist validation and explore methods such as unsupervised or online learning.

## Background

The volume escalation of medical imaging data poses a significant challenge to efficient retrieval processes, especially when combined with inaccurate or incomplete image annotations. The development of integrated web-based viewing systems for pathology digital images  [1] highlights the importance of efficient data storage and distribution in medical imaging research. As related work, our approach presented in  [2] serves as fundament for this research, which integrates instance-level standardized coded descriptors, such as the Systematized Nomenclature of Medicine (SNOMED) CT codes  [3], supplementing the image annotation with DICOM image headers offers a novel approach to enhance the capability to retrieve image-related information. This approach improves the accuracy and comprehensiveness of the retrieved data and addresses the pressing need for efficient management and utilization of vast medical imaging datasets for secondary use.

The Medical Data Integration Center (MeDIC) platform integrates hospital care data for secondary use, including HL7 and medical imaging data. However, there are inconsistencies and inaccuracies in the DICOM header metadata  [4], which hinder effective image categorization and analysis due to varying standards and metadata completeness across different medical imaging systems. In our previous work  [2, 5], we demonstrated the potential of annotating instance-level keywords (modalities, orientation, and anatomic) medical imaging data with publicly available datasets using the Deep Learning (DL) classification method. This method can enhance the retrieval of medical imaging, particularly within the Data Lake infrastructure developed by MeDIC  [6]. However, we encountered a challenge due to the limited availability of publicly accessible datasets that meet the quality and quantity requirements of the Artificial Intelligence (AI) image captioning task. This underscores the need for a clinical image dataset with accurate and standardized annotations, a gap our research aims to fill.

The automatic identification of body parts in medical images has potential applications in improving clinical care, research, and diagnostic support systems. Recent advancements in machine learning, particularly Deep Learning, have significantly enhanced multi-modal and multi-organ body part classification in medical imaging. Researchers have leveraged transfer learning techniques, utilizing pre-trained Convolutional Neural Networks (CNNs) like ResNet  [7] and Inception to enhance computational efficiency and reduce processing time. One study employed a modified CNN architecture, achieving a precision of 82.6% in classifying five organs: lung, liver, heart, kidney, and lumbar spine  [8]. Ma et al.  [9] developed the MedSAM model and trained on the comprehensive COSMOS medical dataset comprising 1.57 million image-mask pairs, covering ten imaging modalities and over 30 cancer types. Additionally, some research has focused on extracting anatomical concepts from radiologist reports, such as Tahmasebi et al.  [10] proposed 56 anatomical concepts derived from SNOMED CT definitions, where 47 classes under body organ structure (SNOMED CT code: 113343008) and nine regional labels. More work needs to be done on classifying medical images using standardized terminologies.One of the few studies in this direction, Filice et al.  [11], who demonstrated promising results by embedding words from the Radiology Gamuts Ontology (RGO) to link diseases and imaging findings with CNNs, achieving an F1 score of 0.66. Despite such efforts, there remains a need for further research into the annotation and retrieval of medical images using standardized terminologies. A standardized approach would facilitate consistent annotation and classification across different imaging modalities, potentially improving interoperability and clinical decision support systems. Despite significant progress, there is room for improvement in classifying medical images by body parts. Ongoing research aims to develop more accurate models, handle diverse imaging modalities, and address the challenges of limited labeled medical data.

### Objective

This paper aims to enhance the DICOM header focusing on X-ray images by generating standardized medical terminologies with Deep Learning. Therefore, we curated a clinical dataset within the University Hospital Schleswig-Holstein (UKSH) with standardized anatomical annotations, including SNOMED CT code and DICOM Body Part Examined Defined Terms. An experienced radiologist provided ground-truth annotations. The curated dataset could enhance the classification performance within the UKSH dataset, thus enhancing the current annotation within MeDIC for secondary use.

We aimed to improve classification performance within the UKSH dataset compared to existing annotation methods and to analyze the potential for enhancing current annotation practices within MeDIC for the secondary use of medical imaging data. We focus on curating a clinical dataset of 20,000 pseudonymized DICOM image series from the UKSH, annotated with standardized anatomical terms, including SNOMED CT codes  [3] and DICOM Body Part Examined Defined Terms. We train, refine, and evaluate Deep Learning models to automatically generate accurate and consistent anatomical annotations for these X-ray images.

This research aims to address the lack of standardized anatomical annotation of X-ray images using Deep Learning for efficient data retrieval and analysis in clinical and research settings. Our study evaluates the effectiveness and potential of Deep Learning in standardizing medical image annotations with a clinical routine dataset. The resulting models will be available to the research community to advance medical image analysis and standardization.

## Methods

**Fig. 1:**
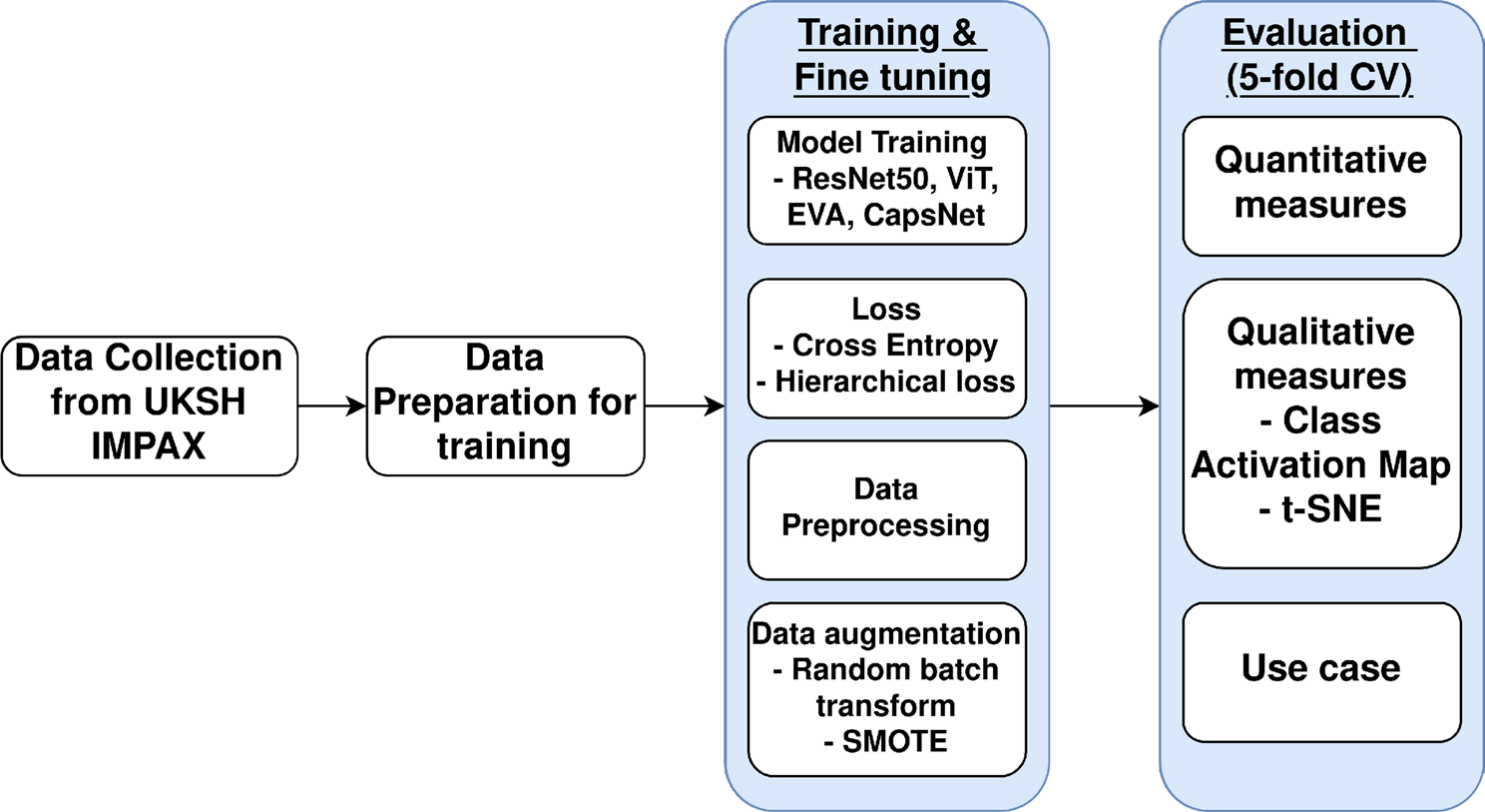
Overview of the training process for the DICOM annotation model. We annotated DICOM images extracted from the pacs system into two classification tasks, trained and evaluated the models for each task

Our study involved acquiring and training 20K real-world clinical X-ray images. Figure 1 shows the overview of the training flow of this study. We standardized the annotation process using 36 SNOMED CT codes, with a radiologist iteratively validating examined (pathological) and visible body parts. We developed and trained DL models to automate this annotation process, achieving high accuracy in classifying examined and visible body parts. Finally, we demonstrated its practical utility through a simulated use-case scenario.

### Data collection & analysis

**Fig. 2:**
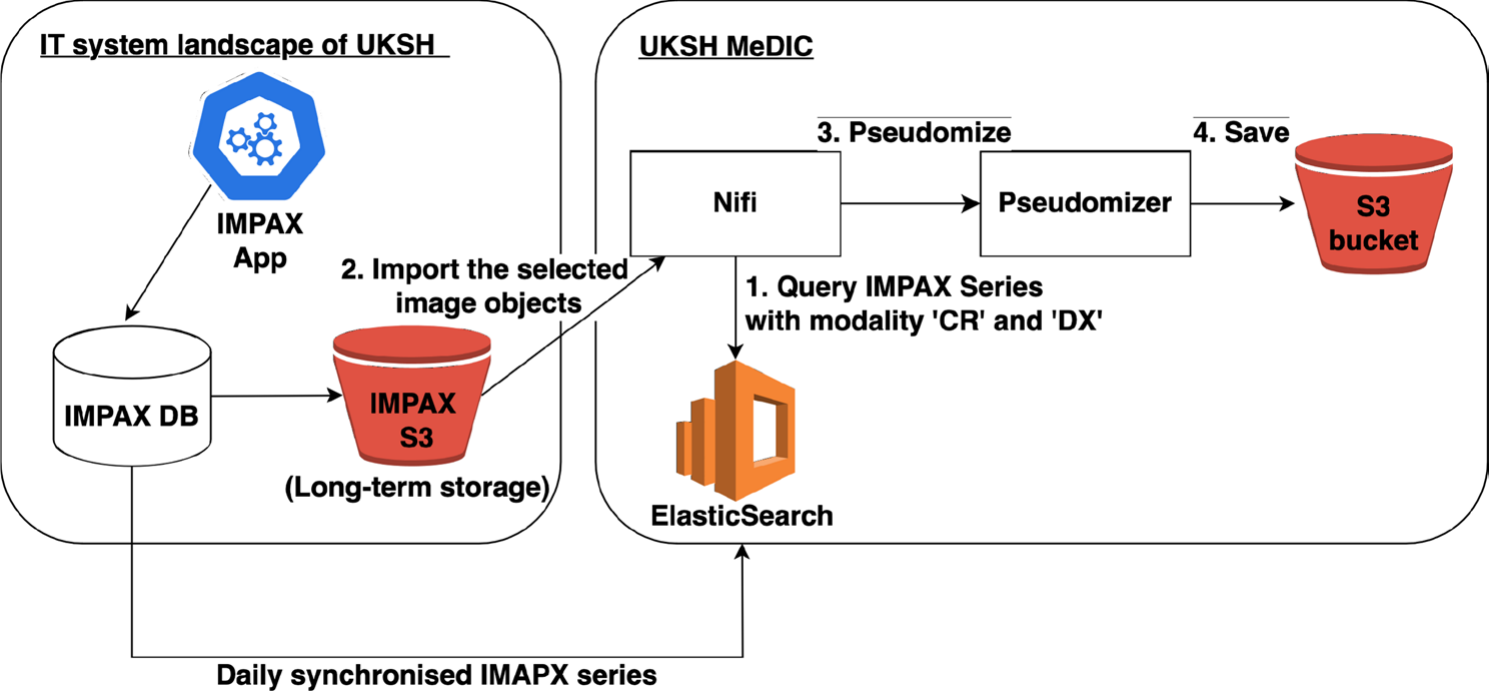
Image data collection flow prior to data preparation and annotation. It started with pacs extraction at UKSH, followed by retrieval through the Datalake’s elasticsearch system and pseudonymization

This study utilized real-world clinical X-ray images from patients at University Hospital Schleswig-Holstein, who provided Broad Consent (BC). These images were routinely captured as part of standard clinical care from Picture Archiving and Communication System (IMPAX) to MeDIC, providing a robust foundation for our research. IMPAX works closely with the Clinical Information System (CIS) to organize radiology reports and store DICOM images in a centralized database. DICOM images are stored in a centralized object storage, ensuring efficient management and retrieval. MeDIC utilized the core components of its Data Lake, namely, Elasticsearch to index DICOM IMPAX Series, NiFi for loading and transporting images, and an S3 bucket for storing the processed images. Figure 2 describes the infrastructure connection of the IMPAX and MeDIC.

We submitted a P2N  [12] application for 20K random pseudonymized X-ray DICOM series to initiate our data acquisition in the training process. We specifically targeted the DICOM series with X-ray modality tags, “Computed Radiography (CR)” and “Digital X-Ray (DX)”, which was obtained from IMPAX. “CR” refers to use a special phosphor plate before scanning and digitizing the image, while “DX” captures images directly through a digital detector. The series was collected as TAR files and stored in an S3 bucket within the MeDIC data lake. Each DICOM file was pseudonymized based on the Application Level Confidentiality Profile  [13].

Our dataset encloses a wide temporal range from the year 2000 to 2023, providing a comprehensive and diverse set of image features crucial for robust deep-learning model training. The collection comprises 20,876 DICOM images, 14,537 “CR”, and 6,339 “DX” modality images. These images were sourced from 1,727 unique patients across 60 different wards. The diverse patient demographics and imaging equipment ensure statistical significance and bias reduction for the Deep Learning training.

**Fig. 3:**
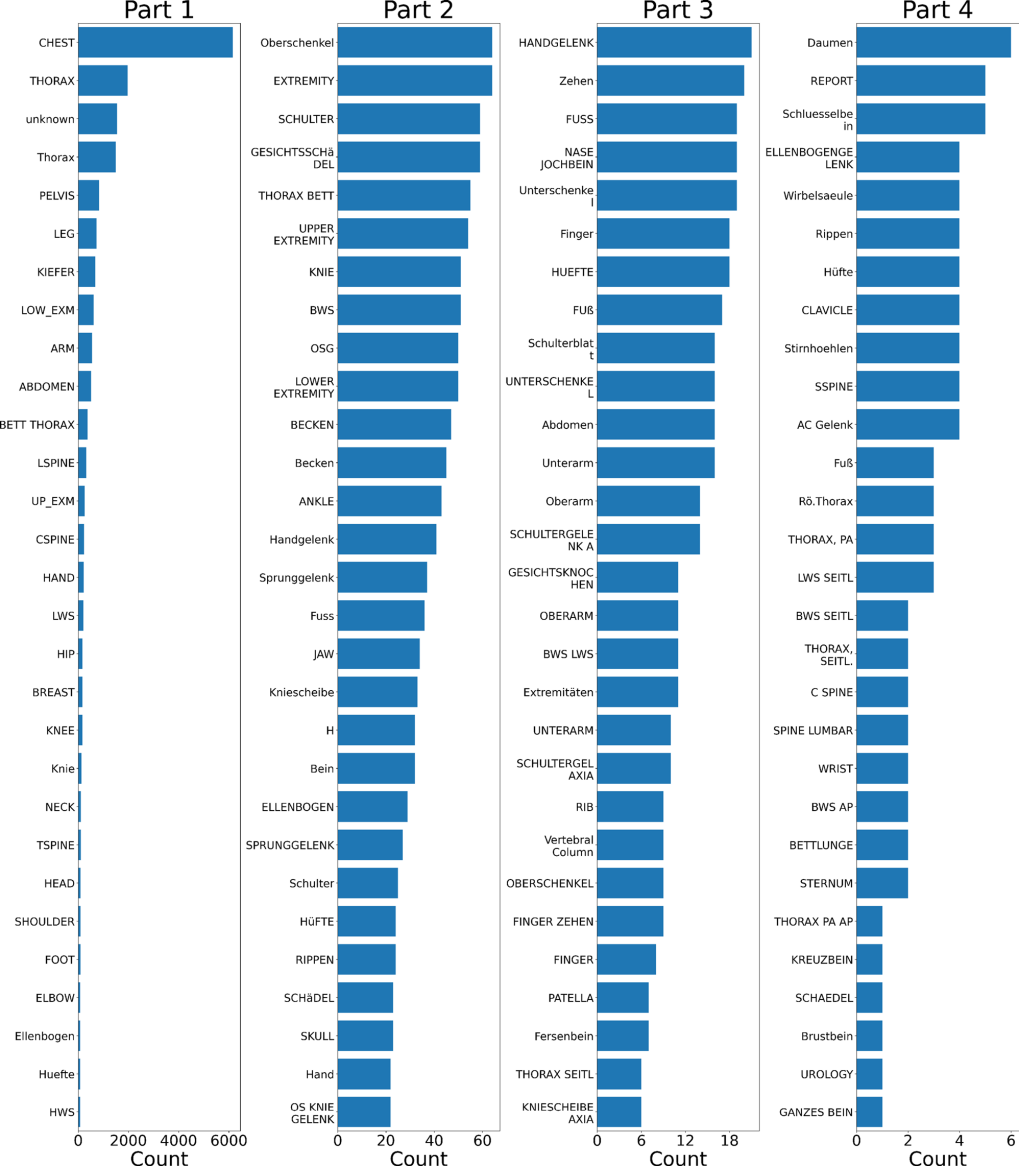
Initial body part distribution in DICOM before radiologist validation (116 classes). Each subplot uses an independent x-axis scale to improve the visibility of minority classes

In our initial analysis, we identified 116 non-standard body parts documented in Fig. 3 across 87% DICOM images and 33 manufacturers from 20,876 images, highlighting the heterogeneity and complexity of the dataset. Moreover, 13% of images miss any description of examined body parts.

The diversity in image sources and temporal range, combined with non-standard annotations and missing data, creates a realistic and challenging dataset. This complexity mirrors the conditions in clinical settings, making our dataset particularly suitable for developing robust and generalizable deep-learning models for medical image annotation.

### Data preparation

**Fig. 4:**
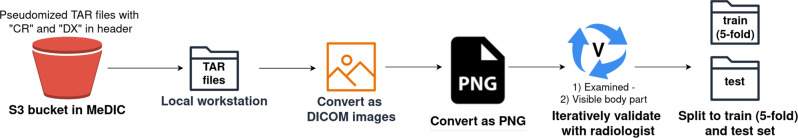
Data preparation process

Figure 4 illustrates the data preparation process. We downloaded the TAR files from S3 to a local workstation with Minio Python library  [14] and extracted files from the TAR archive using the Linux package “atool”, a script for managing file archives of various types. With the Python library Pydicom  [15], we transformed DICOM headers into a CSV file and converted the image content into PNG format. Subsequently, a radiologist iteratively annotated the images with SNOMED CT codes. Finally, we divided the dataset into stratified training (5-fold) and test sets. Through stratified grouping, we randomly shuffled the data while ensuring consistency in class distribution across all folds. This method ensured that each fold contained a representative mix of all classes while maintaining the integrity of the volume data across different partitions.

**Fig. 5:**
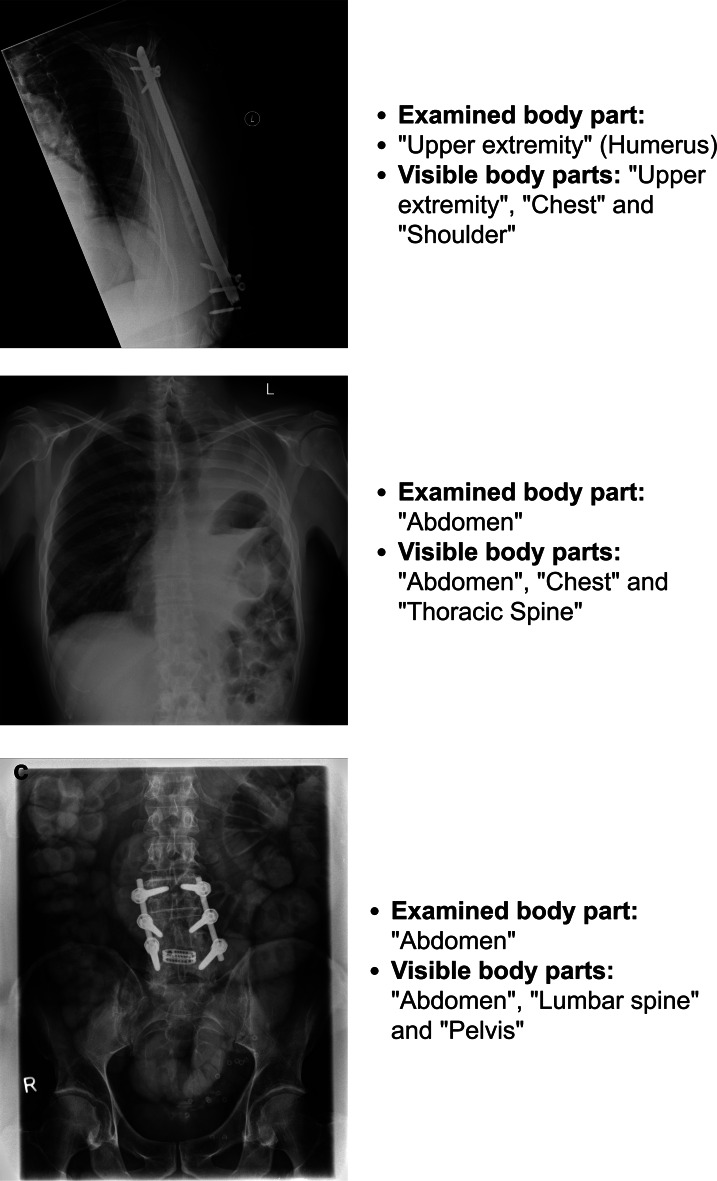
We annotated X-ray images with multiple body part labels in two ways: by indicating the primary pathological findings and by including incidental anatomical structures, thereby highlighting the complexity of multi-label classification in medical imaging. The two annotation types form the foundation for experiments I and II respectively, described in subsequent sections. (**a**) Upper arm fracture with adjacent anatomical structures. (**b**) Abdominal organ displacement with thoracic structures. (**c**) Abdominal radiograph revealing intestinal obstruction with incidental spinal pathologies

We implemented a standardization process using SNOMED CT codes to address the non-standardized labeling. A radiologist with expertise in the field iteratively annotated and validated the images, focusing on two main aspects: I) Examined body parts (tends to be single-label classification), or II) Visible body parts (tends to be multi-label classification). The former method omitted adjacent anatomical structures if they were not the focus of the scan or diagnostic procedure. Some examples were annotated as in Fig. 5. The primary focus of Fig. 5a is the fractured humerus, with adjacent visualization of the chest and shoulder. These additional structures highlight the challenges of multi-label classification in medical imaging. The image 5b primarily illustrates significant displacement of abdominal organs, with additional visible structures including the chest and thoracic spine, emphasizing the multi-region context. The radiograph Fig. 5c shows multiple dilated bowel loops, indicating that intestinal obstruction is the primary pathology. Incidental findings include a lumbar spine prosthesis and the pelvis, providing an an important anatomical context. This image illustrates the importance of comprehensive radiological assessment in identifying both primary pathologies and incidental findings.

A critical step in our annotation process was the standardization of terminologies using DICOM tags and SNOMED CT codes. Table 1 presents the 36 body parts our radiologist employed to annotate the X-ray images. We selected UKSH abbreviations for these body parts based on the most frequently occurring terminologies in our initial DICOM dataset.

**Table 1:** Mapping table of UKSH abbreviation to SNOMED ct

SNOMED	Name	Semantic Types	cui	UKSH abre.
818983003	Abdomen	Body Location or Region	C0000726	ABDOMEN
120574008	Upper extremity part	Body Part, Organ, or Organ Component	C1268196	UP_EXM
85562004	Hand structure	Body Part, Organ, or Organ Component	C0018563	HAND
127949000	Elbow	Body Location or Region	C0013769	ELBOW
8205005	Wrist	Body Location or Region	C0043262	WRIST
7569003	Finger	Body Part, Organ, or Organ Component	C0016129	FINGER
76505004	Thumb structure	Body Part, Organ, or Organ Component	C0040067	DAUMEN
85856004	Acromioclavicular joint structure	Body Space or Junction	C0001208	AC_GELENK
16982005	Shoulder	Body Location or Region	C0037004	SHOULDER
14975008	Forearm	Body Part, Organ, or Organ Component	C0016536	UNTERARM
12921003	Pelvis	Body Part, Organ, or Organ Component	C0030797	PELVIS
302524008	Entire distal femur	Body Part, Organ, or Organ Component	C0448194	UP_LEG
72696002	Knee region structure	Body Part, Organ, or Organ Component	C1963703	KNEE
56459004	Foot	Body Part, Organ, or Organ Component	C0016504	FOOT
29836001	Hip structure	Body Part, Organ, or Organ Component	C0019552	HIP
29707007	Toe	Body Part, Organ, or Organ Component	C0040357	ZEHEN
344001	Ankle	Body Location or Region	C0003086	ANKLE
64234005	Bone structure of patella	Tissue	C0834406	PATELLA
120575009	Lower extremity part	Body Part, Organ, or Organ Component	C1268197	LOW_EXM
76752008	Breast	Body Part, Organ, or Organ Component	C0006141	BREAST
89546000	Bone structure of cranium	Body Part, Organ, or Organ Component	C0037303	HEAD/SKULL
45206002	Bone structure of cranium, Nose	Body Part, Organ, or Organ Component	C0028429	NASE
91609006	Mandible	Body Part, Organ, or Organ Component	C0024687	JAW
51185008	Chest	Body Location or Region	C0817096	CHEST
113197003	Bone structure of rib	Body Part, Organ, or Organ Component	C0035561	RIB
122494005	Cervical spine	Body Part, Organ, or Organ Component	C0728985	CSPINE
122496007	Lumbar spine structure	Body Location or Region	C3887615	LSPINE
264232007	Thoracolumbar	Spatial Concept	C1443270	TLSPINE
122495006	Thoracic spine structure	Body Part, Organ, or Organ Component	C0581269	TSPINE
51282000	Bone structure of spine	Body Part, Organ, or Organ Component	C0549207	SSPINE
51299004	Bone structure of clavicle	Body Part, Organ, or Organ Component	C0008913	CLAVICLE
54735007	Sacrum	Body Part, Organ, or Organ Component	C0036037	KREUZBEIN
56873002	Sternum	Body Part, Organ, or Organ Component	C0038293	STERNUM
−1	unknown			UNKNOWN
−2	report			REPORT

Standardization is essential for two primary reasons: firstly, it provides a consistent framework for characterizing the images and enhances the accuracy and reliability of our annotations. Secondly, it facilitates efficient image retrieval from MeDIC’s Data Lake for secondary use, enabling researchers and clinicians to access relevant images more effectively. By aligning our annotations with established medical terminologies, we ensure that our annotations are internally consistent and compatible with broader medical informatics systems and practices.

### Experiment setting

After preparing the dataset, we set up our experiments based on Table 2, including hardware and DL settings. Since the images were annotated, two experiments were set up based on two main aspects of our radiologist: Experiment I) classifying examined body parts and Experiment II) classifying visible body parts. Within each experiment, our DL setup encompassed a range of models, loss functions, preprocessing techniques to optimize image quality and feature extraction, and data augmentation strategies to enhance model robustness and address class imbalance. The comprehensive setup allowed us to systematically evaluate the impact of different model architectures, loss functions, preprocessing techniques, and data augmentation strategies on the performance of our medical image annotation models.

**Table 2:** Set-up for experiments I and II

PC Hardware & Operating System	
CPU	AMD Ryzen™ 95900X
RAM	Skill DIMM 32 GB DDR4-3200 Kit
GPU	NVIDIA GeForce RTX 960
Operating System	Ubuntu 20.04
**DL experiment settings**	
Model	ResNet50 (Previous work based on IMAGENET1K V2), ViT (IMAGENet-21k), EVA, CapsNet
Loss	Cross-Entropy, custom hierarchical loss
Preprocessing	Resizing, Background removal with OSTU, Top-Bottom Hat and Cartoon-Texture
Augmentation	random batch transformation, random inverted transformation, generated synthetic samples
**Other DL hyperparameters**	
Max Epoch	50 (Early Stopping patience = 8)
Batch	64
Shuffle	True
Fine-tuning	stratified 5-fold grid search cross validation

#### Choosing a DL model architecture

As a foundation for advanced transfer learning, we leveraged the fine-tuned pre-trained ResNet50 architecture from our prior research  [2]. Furthermore, we expanded our investigation to encompass a range of more state-of-the-art models, such as Vision Transformer (ViT)  [16], Efficient Vision Transformers (Eva)  [17], and Capsule Networks (CapsNet)  [18].

The Vision Transformer (ViT) is a neural network architecture that applies the transformer model, initially developed for natural language processing, to image recognition tasks by dividing images into patches and processing them as sequences. It achieves impressive performance on large datasets  [16].

Efficient Vision Transformers (Eva) aim to make transformer models more efficient and suitable for deployment in resource-constrained environments by optimizing the architecture to reduce computational complexity and enhance scalability  [17].

Zhang et al.  [18] introduced Capsule Networks (CapsNet) to address the limitations of traditional CNNs. CapsNet uses capsules—vectors of neurons that encode spatial hierarchies and relationships through dynamic routing. Unlike traditional patch-based approaches, CapsNets incorporate global context information, making them more robust in object orientation and providing more interpretable representations. However, this approach comes with higher computational costs.

#### Custom loss functions with three-level hierarchical structure

We proposed a custom hierarchical loss function, as outlined in Table 3, to penalize incorrect hierarchical predictions more heavily and reduce unfair evaluations. We organized the 36 body parts in Table 1 into a three-level hierarchical structure. Level 1 represents the broadest body regions in this hierarchy, while Level 3 represents the specific sub-regions within these broader categories. This hierarchy allows us to structure the hierarchical loss function to reflect the severity of errors based on their level. For example, misclassifying a hand as a finger or upper extremity should incur a more minor penalty than misclassifying it as an abdomen.

**Table 3:** Custom hierarchical loss within the three-level body region hierarchy

Level1	Level2	Level3
Cranium	Cranium	Cranium
Cranium	Cranium	Nose
Cranium	Cranium	Mandible
Abdomen	Abdomen	Abdomen
Chest	Breast	Breast
Chest	Chest	Chest
Chest	Clavicle	Clavicle
Chest	Rib	Rib
Chest	Sternum	Sternum
Spine	Spine	Spine
Spine	Cervical spine	Cervical spine
Spine	Thoracic spine	Thoracic spine
Spine	Lumbar spine	Lumbar spine
Spine	Thoracolumbar	Thoracolumbar
Unknown	Unknown	Unknown
Unknown	Report	Report
Upper extremity	Upper extremity	Upper extremity
Upper extremity	Shoulder	Shoulder
Upper extremity	Shoulder	Acromioclavicular joint
Upper extremity	Elbow	Elbow
Upper extremity	Forearm	Forearm
Upper extremity	Hand	Hand
Upper extremity	Hand	Wrist
Upper extremity	Hand	Finger
Upper extremity	Hand	Thumb
Lower Extremity	Lower Extremity	Lower Extremity
Lower Extremity	Pelvis	Pelvis
Lower Extremity	Pelvis	Sacrum
Lower Extremity	Pelvis	Hip
Lower Extremity	Distal femur	Distal femur
Lower Extremity	Knee	Knee
Lower Extremity	Knee	Patella
Lower Extremity	Ankle	Ankle
Lower Extremity	Foot	Foot
Lower Extremity	Foot	Toe

To quantify the error at each hierarchical row, we defined a layer-specific loss using binary cross-entropy, represented as:

$$lloss_n = -\sum_{c=1}^N y_{o,c}\log(p_{o,c})$$

where *N* is the number of unique classes in that level, *y* is the ground truth binary indicator (0 or 1) if class label *c* is the correct classification for observation *o*, and *p* is the predicted probability for class *c*. The overall loss function, $J(\theta)$, is then formulated as a weighted sum of the losses at each hierarchical level. This approach ensures that errors at higher levels of the hierarchy (broader categories) are penalized differently than those at finer levels (specific sub-regions). The total loss is calculated as follows:

$$J(\theta) = 0.4 \cdot lloss_1 + 0.3 \cdot lloss_2 + 0.3 \cdot lloss_3$$

where *lloss*_1_, *lloss*_2_, and *lloss*_3_ are the binary cross-entropy losses at levels 1, 2, and 3 of the hierarchy. We chose the weights (0.4, 0.3, and 0.3) to balance the influence of errors at different levels, giving slightly more importance to the broadest categorization errors while still considering the finer distinctions.

#### Three preprocessing strategies for model optimization

We systematically evaluated various image preprocessing techniques in conjunction with the best-performing model architecture and loss function from each experiment to optimize our model performance. Our preprocessing procedure comprised three alternatives: 1) Background detection and removal, 2) Contrast enhancement using Top-bottom hat transformation, and 3) Image decomposition with Cartoon-Texture (Cartex).

**Background detection and removal** Background detection and removal are crucial for enhancing the performance, accuracy, and efficiency of computer vision tasks. By simplifying images and allowing models to focus on the most relevant features, this technique significantly improves the outcomes of various applications  [19].

We implemented thresholding as a computationally efficient method to separate foreground and background in grayscale images. By simply choosing a threshold value, all pixels with intensity values below the threshold were assigned to one binary class “0”, while those above the threshold were assigned to the other binary class “1”, resulting in binary images. Specifically, we employed Otsu’s method (OTSU)  [20] for automatic image thresholding, determining an optimal threshold by maximizing the between-class variance:

$$\mathrm{var}(T) = P_0(T) \cdot P_1(T) \cdot (m_0(T) - m_1(T))^2$$

Here, $P_0(T)$ and $P_1(T)$ represent the probabilities of the background and foreground regions, respectively, and $m_0(T)$ and $m_1(T)$ are their respective mean grayscale intensity values. While Otsu’s method is effective, it has limitations with noisy images, uneven illumination, objects close to background intensity, and small objects, as it is a global thresholding algorithm.

**Top-bottom hat and Cartoon-Texture in previous work** Building on our last work  [2], we incorporated contrast enhancement using the Top-bottom hat transformation and image decomposition with Cartoon-Texture (Cartex). Our earlier studies demonstrated that these techniques can significantly enhance model performance in medical image classification tasks.

By systematically comparing these preprocessing techniques, we aimed to determine the optimal combination to maximize our model’s ability to accurately annotate and classify medical images.

#### Data augmentation: random transform, invert, and SMOTE

Data augmentation is a technique that enhances model performance by generating new, artificial data derived from existing training samples  [21]. Our study implemented three different augmentation strategies: random batch transformation, X-ray image inversion, and tail class augmentation using the Synthetic Minority Oversampling Technique (SMOTE).

**Random batch transformation** As in our previous work, we applied random batch transformation with standard data augmentation techniques. The parameters, as outlined in  [21]: We applied Dihedral transform  [22] with a probability of 0.5. With a probability of 0.75, we used a random rotation of additional 10 degrees. With a probability of 0.75, a random zoom between a scale of 0.9 and 1.1, and a perspective warping of 0.2. With a probability of 0.75, we applied a change in brightness, and contrast of a maximum scale of 0.2 was applied. We applied a random resize crop by sampling a scale between 0.9 and 1.0, and subsequently resized the cropped image to the final image size.

**Inverting X-ray images** The background of a standard X-ray image is usually black, representing the areas where X-rays have passed through without being absorbed by the body. In an inverted X-ray image, the background is white or light. Given the diverse color schemes of X-ray images in the PACS dataset  [23], radiologists often invert the colors of an X-ray for several reasons, primarily to improve the clarity and detail of the image, thereby enhancing diagnostic accuracy. Inverting the colors can make specific structures or pathological conditions more visible. Subtle fractures, small lesions, or fine details in soft tissues may become more apparent against a different background.

We applied random inversions to each batch with a 0.5 probability, ensuring a balanced representation of standard and inverted X-ray images in our training data.

**SMOTE** To address the class imbalance and ensure uniform distribution across all categories, we generated synthetic samples for tail classes with the SMOTE. This oversampling method is particularly effective for learning from imbalanced datasets  [24]. Fine-tuning the model with augmented tail class data ensured better generalization across all categories, ultimately leading to a more balanced and accurate model overall.

This comprehensive augmentation strategy, combining random transformations, image inversion, and synthetic sample generation, significantly contributed to the model’s ability to learn from a diverse dataset.

### Experimental evaluation

**Fig. 6:**
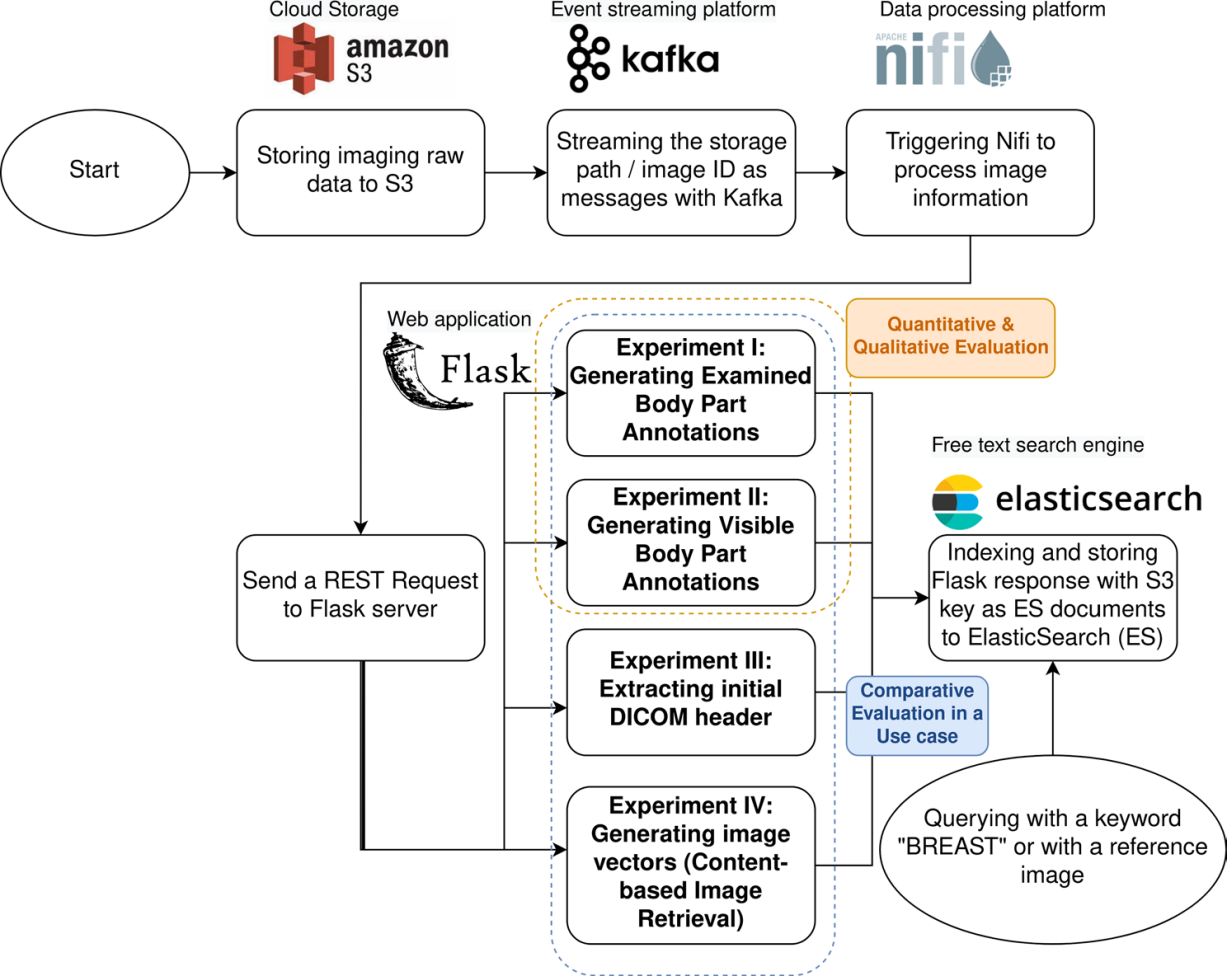
Image annotation in use case. Our DL-based classification, quantitative- and qualitative evaluation were focused on experiment I and II. For a comparative evaluation, we conducted four annotation methods (experiment I to IV) in a simulated real-world scenario

As illustrated in Fig. 6, we employed a range of strategies—quantitative metrics, visualization techniques, and interpretability tools—to evaluate the performance of our DL multi-label classification model in Experiments I and II. For comparative evaluation and to ensure generalization, we tested the retrieval capability of our model against two additional methods, original DICOM headers and content-based, in a possible use-case scenario with our proposed image pipeline  [5], resulting in a total of four experiments (Experiments I to IV).

#### Quantitative measures

Our DL-based classification and quantitative evaluation were focused on Experiment I and II. For each experiment, we used standard evaluation metrics  [25], such as Accuracy, Precision, Recall, and F1-Score, to quantify the model’s performance across all labels to identify our best model. Precision provides insights into the model’s ability to identify relevant labels correctly. Recall measures the model’s effectiveness in capturing all relevant labels. F1-Score balances between precision and recall, offering a single metric for model performance.

#### Qualitative measures of best models

After stratified 5-fold cross-validation with grid search, we visualized the best model ability with t-distributed Stochastic Neighbor Embedding (t-SNE)  [26] and Multi-Label Confusion Matrix. t-SNE reduces the high-dimensional feature space learned by our model into a two-dimensional space. By projecting the feature representations into a two-dimensional space, we evaluate how efficient the model clusters different labels and identifies the underlying patterns or overlaps. This visualization aids in assessing the model’s ability to distinguish between different label combinations in a multi-label setting  [26]. Additionally, we generated multi-label confusion matrices to analyze the model’s performance on individual labels and their interactions. This matrix captures true positives (TP), false positives (FP), true negatives (TN), and false negatives (FN) for each label, providing detailed insights into the model’s strengths and weaknesses  [27]. By examining the confusion matrix, we can identify labels often confused with others and understand the model error patterns.

We utilized Class Activation Maps (CAMs)  [28] to interpret the regions of the input images that our model focused on while making predictions. CAM provides a heatmap overlay on the original image, highlighting the most influential areas for predicting each label. This interpretability tool helps us verify if the model is attending to the correct regions for each label, ensuring that the model’s decision-making process is transparent and aligned with human intuition.

#### Comparative evaluation in a practical use case

For a comparative evaluation, we conducted four annotation methods—Experiments I and II as detailed in the previous sections, along with additional Experiments III and IV—in a simulated real-world scenario. These annotation methods were then evaluated using our proposed image annotation pipeline  [5]:

- Experiment I) Examined body part annotations (refer to Annotation 1 in 5a)
- Experiment II) Visible body part annotations (refer to Annotation 2 in 5a)
- Experiment III) Initial DICOM tags
- Experiment IV) Image vector extracted from a pre-trained ResNet50 (IMAGENET1K V2)

In Experiments III, the annotations were derived from the initial UKSH DICOM tags “Body Part Examined (0018,0015)”, extracted using the Python Library Pydicom  [15] as in Sect. 2.2. In Experiment IV, the images features were processed with a pre-trained ResNet50 (IMAGENET1K V2). The model was pre-trained on the comprehensive ImageNet dataset, which contains over a million images across a thousand categories. This pre-training enables ResNet50 to extract rich and informative features from medical images effectively, making it a valuable comparison to our proposed method within the context of our study.

All the components in the pipeline of Fig. 6 were open-source projects, had active communities, and were widely used in various projects. After storing the raw images in S3, the data processing platform Nifi streamlined the images via Kafka and sent the image paths to a Python web server, which then annotated the images. We compared the annotation results from our two DL image classifiers against those obtained from initial DICOM tags in PACS and Content-based Image Retrieval (CBIR) techniques in four experiments. All the generated annotations or image vectors were indexed in ElasticSearch for the later query, with a keyword or a reference image. After the automated annotation, the images were moved to another bucket within S3.

In our simulated scenario, researchers were tasked with retrieving X-ray breast images, from the MeDIC database rather than from KIS or PACS systems. This setup is particularly relevant given the increasing focus on AI-assisted breast cancer detection in radiology  [29]. For retrieving in Experiment I to III, we employed a keyword-based search in ElasticSearch using the class label “BREAST”, a method similar to that described by Safaei et al.  [30]. To identify the most similar images based on image vectors in Experiment IV, we utilized cosine similarity, a technique widely used in medical image retrieval systems  [31].

By combining quantitative, qualitative, and evaluation strategies, we ensured a thorough assessment of our DL multi-label classification model, enabling us to understand its performance, interpretability, and potential areas for improvement.

## Result

The curated dataset was annotated based on two main aspects: 1) Examined Body Parts and 2) Visible Body Parts. These aspects are illustrated in Fig. 5 respectively. Two experiments, detailed in Sects. 3.2 and 3.3, were set up and evaluated.

### Dataset: simplifying 116 non-standard labels into 36 SNOMED CT codes

**Fig. 7:**
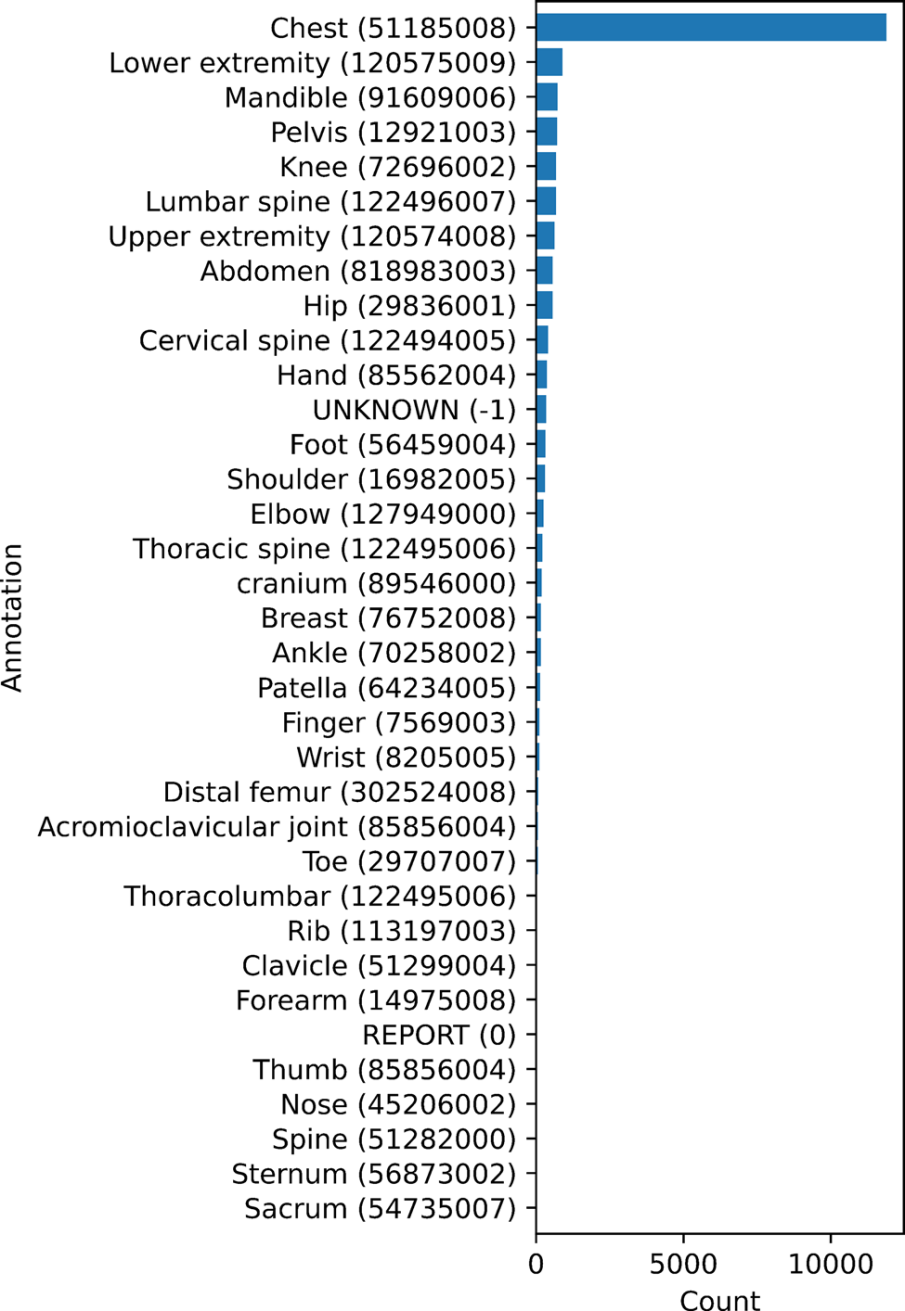
Body part annotation after radiologist validation

With validation provided by an experienced radiologist, we used only 36 relevant SNOMED CT codes, significantly reducing the complexity from the original 116 non-standard body part descriptions in Fig. 3. The distribution is shown in Fig. 7.

### Performance of experiment I: examined body part classification

This section focuses on Examined Body Part Classification (as Annotation 1 in Fig. 5a). We trained the dataset with different combinations based on Table 2.

Table 4 presents the comparison of pre-trained models with different loss functions. The models evaluated were ResNet-50, ViT, EVA, and CapsNet. While ResNet-50, ViT, and EVA were trained with standard multi-label loss functions, CapsNet utilized a custom multi-label loss due to its unique use of capsules and vectors, which require specialized loss functions to handle their representations. Additionally, ResNet-50 was also trained using the custom hierarchical loss in Section 2.3.2 to explore its training penalty to the anatomical hierarchy.

**Table 4:** Comparison of model architectures and loss functions

Model	ResNet50	ResNet50	ViT	EVA	CapsNet
Loss	Cross entropy	Custom hierarchical	Cross entropy	Cross entropy	Custom multi-label
Accuracy	0.887	**0.889**	0.832	0.810	0.535
Precision	0.562	**0.576**	0.486	0.562	0.314
F1 score	0.595	**0.607**	0.528	0.591	0.297
Recall	0.676	**0.677**	0.627	0.674	0.324

Regarding performance shown in Table 4, ResNet50 with custom hierarchical loss shows the highest values across accuracy (0.889), precision (0.576), F1 score (0.607), and recall (0.677). ViT follows ResNet50 with slightly lower values across these metrics, with an accuracy of 0.832. CapsNet demonstrates the lowest performance metrics among the models listed, with an accuracy of 0.535.

After selecting the model architecture and loss function, we compared the image pre-processing algorithms, as shown in Table 5, resizing achieves the highest accuracy (0.887), followed closely by Top-Bottom Hat (0.743). The background removal with OTSU threshold has the lowest accuracy (0.737).

**Table 5:** Comparison of preprocessing methods

Resizing	$\checkmark$	$\checkmark$	$\checkmark$	$\checkmark$	$\checkmark$
Background removal with OTSU		$\checkmark$			
Top-Bottom Hat			$\checkmark$		$\checkmark$
Cartex				$\checkmark$	$\checkmark$
Accuracy	**0.889**	0.745	0.737	0.743	0.734
Precision	**0.576**	0.571	0.563	0.577	0.563
F1 score	**0.607**	0.600	0.582	0.608	0.598
Recall	**0.677**	0.677	0.639	0.665	0.671

Top-Bottom Hat achieves the highest precision (0.577), followed by resizing (0.571). Removing background with OTSU threshold, and Cartex has the same precision score (0.563), the lowest among the methods.

In terms of the F1 Score, the Cartex has the highest score (0.608), followed closely by resizing (0.607). Remove margin with OTSU threshold has the lowest score (0.582).

Regarding recall, resizing has the highest recall score (0.677), followed by Cartex (0.671). Background removal with the OTSU threshold has the lowest recall score (0.639).

Only resizing generally performs the best across all metrics except Precision, which is still competitive. Cartex has the highest F1 score. Top-Bottom Hat consistently shows lower performance across Accuracy, Precision, F1 score, and Recall. The choice of preprocessing methods (resizing, background removal, top-bottom hat, Cartex) significantly affects performance metrics, with each method contributing differently to overall effectiveness.

Table 6 presents the results of various augmentation techniques applied to our dataset with pathological annotation and their impact on performance metrics. Column 1, which doesn’t employ a specific augmentation method, has the highest accuracy (0.889) but lower precision (0.576) and F1 score (0.607) compared to columns 3 and 4. Column 3, with only SMOTE, shows a balanced performance with moderate scores in accuracy (0.754), precision (0.602), F1 score (0.620), and recall (0.667). Column 4 shows the best precision (0.613), F1 score (0.643), and recall (0.731), indicating that it could be a promising technique for optimizing overall model performance.

**Table 6:** Comparison of augmentation methods

	Column 1	Column 2	Column 3	Column 4
Random inverted batch transformation		$\checkmark$		$\checkmark$
Random batch transformation		$\checkmark$		$\checkmark$
Generate synthetic samples with SMOTE			$\checkmark$	$\checkmark$
Accuracy	**0.889**	0.738	0.754	0.787
Precision	**0.576**	0.514	0.602	0.613
F1 score	**0.607**	0.541	0.620	0.643
Recall	**0.677**	0.663	0.667	0.731

This analysis highlights the potential of combining SMOTE and other augmentation algorithms to improve the performance of a classification model across multiple evaluation metrics.

After fine-tuning and grid-search cross-validation, our best model achieved 0.889 of accuracy, 0.576 of precision, 0.730 of F1 score, and 0.676 of recall.

#### Visualization of model attention via CAM

**Fig. 8:**
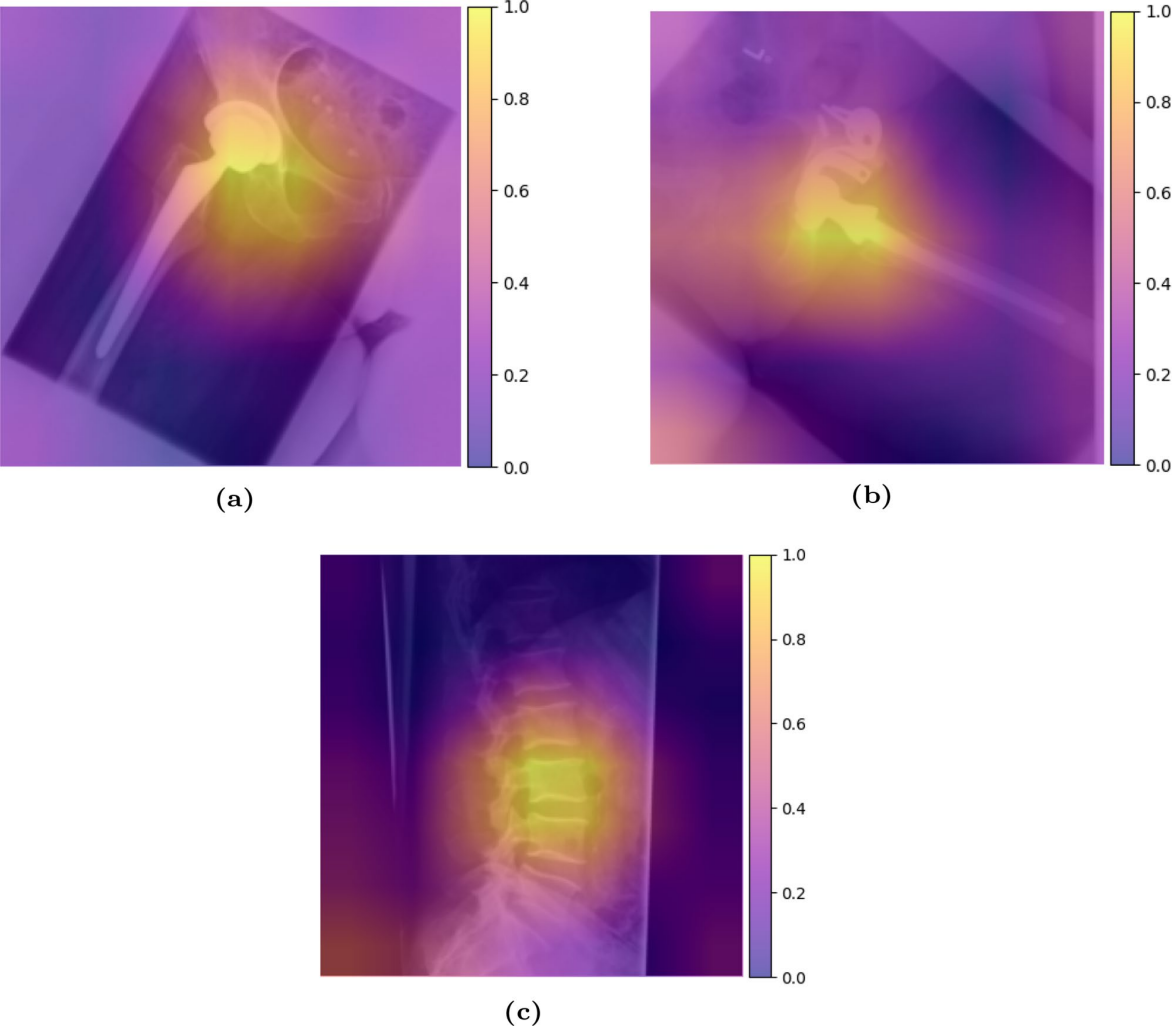
Cam of top 3 most confused test images. (**a**) True label = low extremity; prediction = hip. (**b**) True label = pelvis; prediction = hip. Black borders appearing on rescan X-rays may be caused by unexposed film edges, improper scanner alignment, or spacers used during the digitisation process. c) true label = thoracic spine; prediction = lumbar spine

Figure 8 presents CAMs for the top confused predictions, corresponding to the highest misclassification loss. This shows that the classifier learned the ROI correctly but was still confused with the intrinsic division of certain classes.

### Performance of experiment II: visible body part classification

This section focuses on Visible Body Part Classification (as Annotations of visible body parts in Fig. 5) and aims to compare performance across model architectures following the setup in Table 2, and to evaluate how preprocessing and augmentation influence the balance between accuracy and metric stability.

Table 7 summarizes the capabilities and performance metrics for five different models. The pretrained ResNet50 models, regardless of the loss function used, consistently outperformed the other models across all metrics. The ResNet50 model with Cross Entropy loss generally had the highest accuracy (0.853), precision, and F1 score, while the Custom Hierarchical loss version achieved the highest recall(0.743). ViT and EVA had slightly lower performance metrics compared to ResNet50. CapsNet, using a Custom Multi-label loss, had the lowest performance across all evaluated metrics.

**Table 7:** Comparison of model architectures and loss functions

Model	ResNet50	ResNet50	ViT	EVA	CapsNet
Loss	Cross entropy	Custom hierarchical	Cross entropy	Cross entropy	Custom multi-label
Accuracy	**0.853**	0.850	0.808	0.810	0.531
Precision	**0.642**	0.636	0.570	0.562	0.433
F1 score	**0.674**	0.672	0.612	0.591	0.347
Recall	**0.730**	0.743	0.730	0.674	0.472

Continuing training with the best model architecture and loss function, the ResNet50 model with Cross Entropy loss, in Table 8 preprocessing with solely resizing performed best in accuracy, precision, F1 score, and recall. Background removal with the OTSU feature showed the lowest performance across all metrics.

**Table 8:** Comparison of preprocessing methods

Resizing	$\checkmark$	$\checkmark$	$\checkmark$	$\checkmark$	$\checkmark$
Background removal with OTSU		$\checkmark$			
Top-Bottom Hat			$\checkmark$		$\checkmark$
Cartex				$\checkmark$	$\checkmark$
Accuracy	**0.853**	0.842	0.850	0.852	0.850
Precision	**0.642**	0.633	0.641	0.633	0.634
F1 score	**0.674**	0.663	0.671	0.665	0.667
Recall	**0.730**	0.714	0.722	0.722	0.724

In Table 9, the highest Precision (0.650) and highest F1 score (0.682) is achieved by Column 3, which didn’t apply any augmentation methods. However, the highest Accuracy (0.856) is achieved by Column 4, which uses random batch transformation, random inverted transformation, and synthetic samples with SMOTE.

**Table 9:** Comparison of augmentation methods

	Column 1	Column 2	Column 3	Column 4
Random inverted batch transformation		$\checkmark$		$\checkmark$
Random batch transformation		$\checkmark$		$\checkmark$
Generate synthetic samples with SMOTE			$\checkmark$	$\checkmark$
Accuracy	0.853	0.852	**0.853**	0.856
Precision	0.642	0.609	**0.650**	0.596
F1 score	0.674	0.650	**0.682**	0.628
Recall	0.730	0.732	**0.757**	0.696

#### t-distributed stochastic neighbor embedding (t-SNE) and confusion matrix

**Fig. 9:**
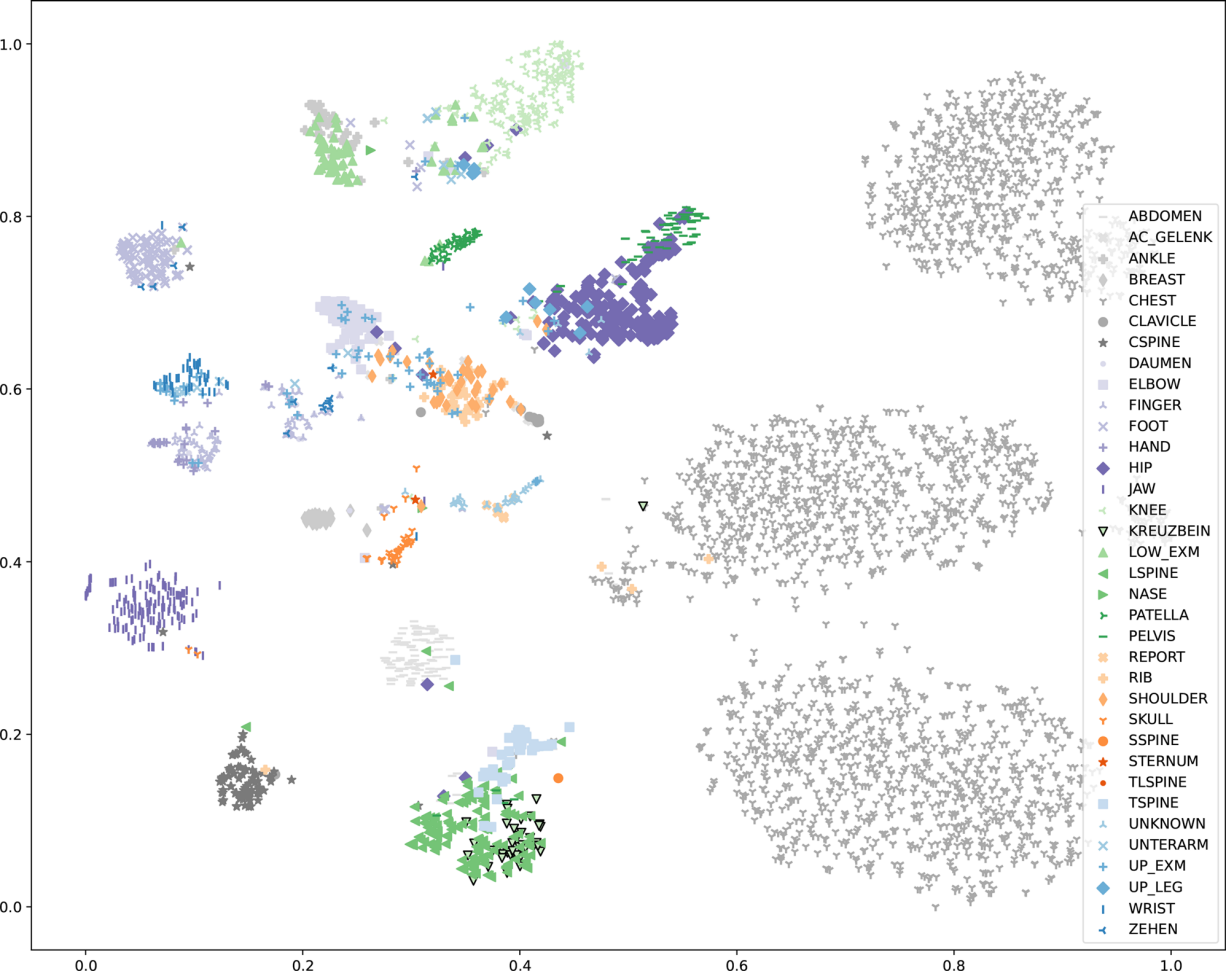
A t-distributed stochastic neighbor embedding (t-SNE) reduces the high dimensional feature space to low-dimensional embeddings. The x- and y-axes correspond to the first and second dimensions of the low-dimensional t-SNE embedding

Figure 9 shows the t-distributed stochastic neighbor embedding (t-SNE). Most of the data points of each class were grouped in the two-dimensional map, showing that the classifier could classify these images correctly.

**Fig. 10:**
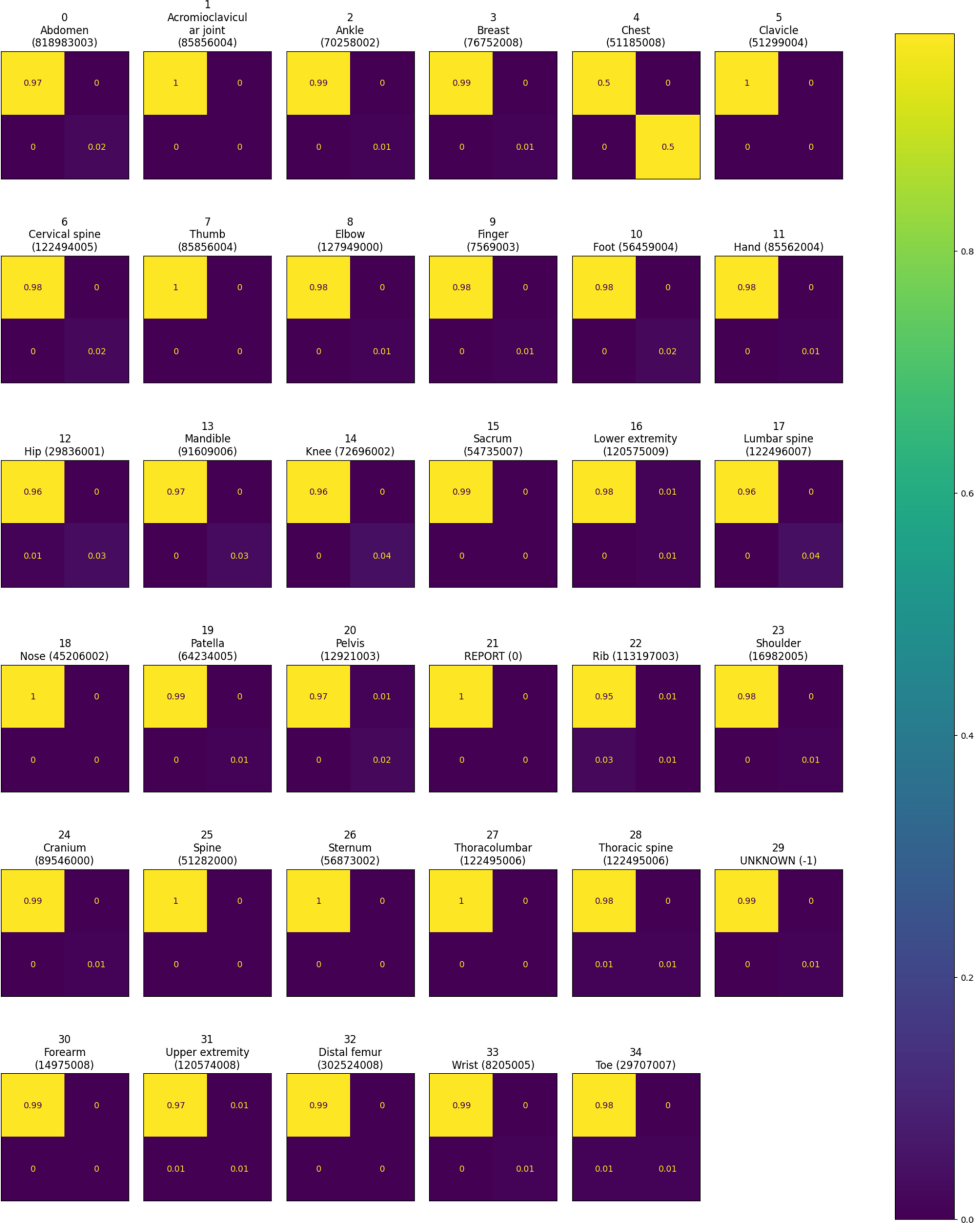
Multi-label confusion matrix showing true versus predicted labels across all classes, highlighting model performance and misclassifications in an imbalanced dataset

Figure 10 shows the multi-label confusion matrix. The most confusing classes were “Sacrum” (SNOMED code: 54735007), “Spine” (SNOMED code: 51282000), “Sternum” (SNOMED code: 56873002) and leaf classes of extremities. Following the analysis of the confusion matrix, Table 10 presents the detailed classification report for each class in the dataset, summarizing key performance metrics including precision, recall, F1-score, and support.

**Table 10:** Per-class classification report with precision, recall, F1-score, and support. Classes with precision and recall values between 0.9 and 1.0 (shown in bold) demonstrate remarkable predictive performance, indicating reliable discrimination. In contrast, other classes reveal areas for potential improvement

Class	Precision	Recall	F1-score	Support
Abdomen (818983003)	0.94	0.89	0.92	116
Acromioclavicular joint (85856004)	0.6	0.3	0.4	10
Ankle (70258002)	0.75	0.75	0.75	48
**Breast (76752008)**	0.97	0.97	0.97	33
**Chest (51185008)**	1.0	1.0	1.0	2382
Clavicle (51299004)	0.9	0.82	0.86	11
**Cervical spine (122494005)**	0.98	0.9	0.93	88
Thumb (85856004)	0.0	0.0	0.0	9
Elbow (127949000)	0.87	0.82	0.85	67
Finger (7569003)	0.81	0.75	0.78	87
Foot (56459004)	0.93	0.88	0.9	91
Hand (85562004)	0.87	0.93	0.9	74
Hip (29836001)	0.89	0.78	0.83	171
**Mandible (91609006)**	0.96	0.93	0.95	158
**Knee (72696002)**	0.97	0.93	0.95	180
Sacrum (54735007)	0.86	0.45	0.59	42
Lower extremity (120575009)	0.74	0.86	0.8	81
Lumbar spine (122496007)	0.91	0.89	0.9	194
Nose (45206002)	0.5	0.5	0.5	2
**Patella (64234005)**	0.95	0.93	0.94	44
Pelvis (12921003)	0.68	0.85	0.76	86
REPORT (0)	1.0	0.43	0.6	7
Rib (113197003)	0.48	0.25	0.33	171
Shoulder (16982005)	0.74	0.75	0.74	83
Cranium (89546000)	0.94	0.82	0.87	38
Spine (51282000)	0.0	0.0	0.0	1
Sternum (56873002)	0.0	0.0	0.0	5
Thoracolumbar (122495006)	0.0	0.0	0.0	10
Thoracic spine (122495006)	0.87	0.44	0.58	102
UNKNOWN (−1)	0.85	0.88	0.87	51
Forearm (14975008)	0.67	0.4	0.5	20
Upper extremity (120574008)	0.65	0.54	0.59	100
Distal femur (302524008)	0.58	0.49	0.53	39
Wrist (8205005)	0.84	0.7	0.77	54
Toe (29707007)	0.86	0.64	0.74	76

### Breast image use case

**Fig. 11:**
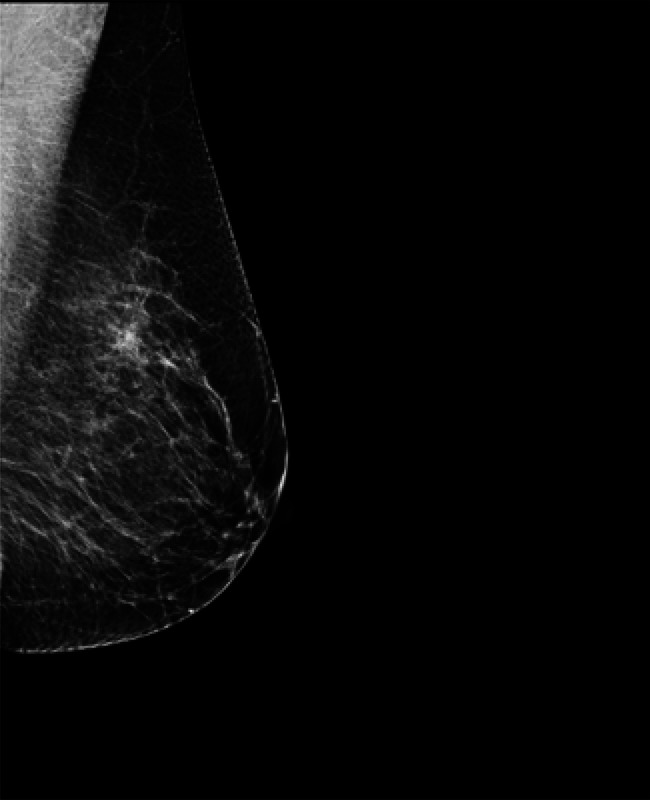
Reference image of “breast”

We implemented the proposed image pipeline using a dataset of 20K DICOM images. By searching with the keyword “Breast”, we retrieved a specific number of images for different experiments: in Experiment I, we found 167 images with pathological image annotations; in Experiment II, 165 images with visible body part annotations; and in Experiment III, 169 images with relevant DICOM headers. Additionally, using pre-trained ResNet50 reference image vectors of Fig. 11 for Experiment IV, we obtained 0 images with a similarity score greater than 0.55 and 14 images with a similarity score greater than 0.3.

**Fig. 12:**
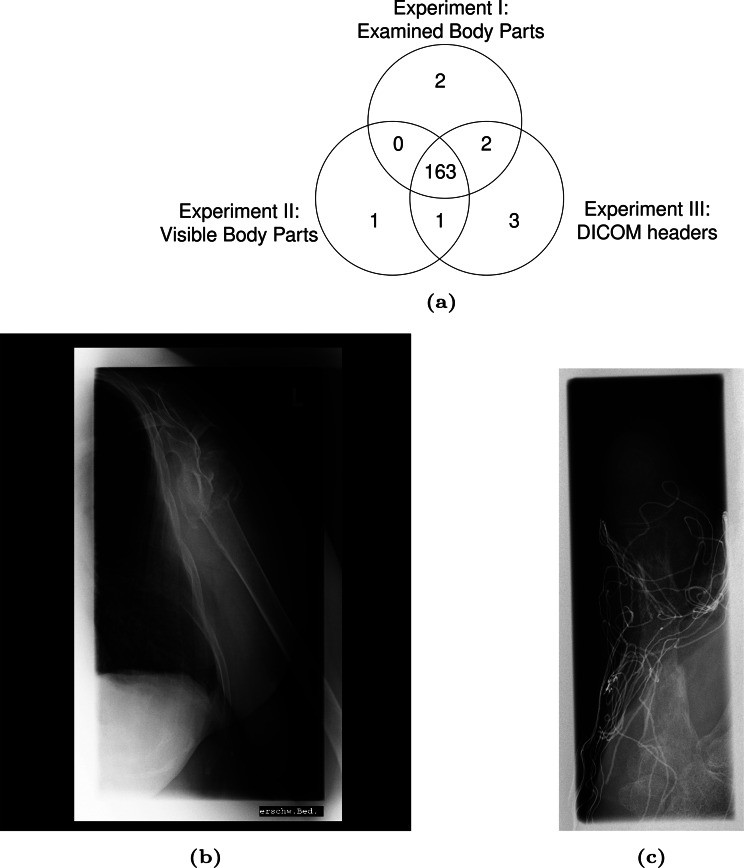
The intersection of dl annotation and DICOM header achieved 100% accuracy by complementing each other (**a**) Venn diagram of use case result. (**b**) Example of DICOM header mislabeling corrected by deep learning prediction. The original DICOM header labeled the image as “breast” incorrectly, while the deep learning model correctly classified it as “upper extremity.” (**c**) Example of misclassification. The DICOM header correctly identifies the image as a “hand” X-ray, whereas the deep learning model incorrectly predicts the class as “breast”

As shown in Fig. 12 and Table 10, the intersection of Experiment I and Experiment III achieved the best result, with an accuracy of 100% and a recall of 98.7% across 165 images. The DL model wrongly predicted two images based on the DICOM headers, and DICOM images misclassified three. By correcting these errors through the integration of the original DICOM headers and DL-generated predictions, we significantly enhanced the overall accuracy of our use case. Experiment II and III intersection obtained 164 images, just one breast image less. In Experiment IV, the content-based image retrieval struggled to find an optimal dataset, retrieving fewer than fifteen breast images. This suggests that directly using the text-based retrieval method is more effective.

## Dissussion

To our knowledge, our study demonstrates the first successful application of deep learning for the automated standardization of X-ray images to SNOMED CT codes. The models were trained with 20k real clinical DICOM images and thoroughly evaluated using diverse Deep Learning techniques. This pioneering approach showcases the potential of DL to optimize medical image annotations and retrieval for secondary use in MeDIC. The results in Table 4 indicate that ResNet50 outperformed ViT and CapsNet across all evaluated metrics. The choice of loss function was observed to influence model performance, with the custom hierarchical loss contributing to improved results compared to standard cross-entropy in this evaluation.

The DL model for X-ray body part classification achieved an accuracy of 0.889, demonstrating reliability in the automated annotation process across varying image qualities and patient demographics. Notably, 116 diverse DICOM tags and 13% of the images needed a description of examined body parts in our initial DICOM analysis. This non-standard and absence of labeling in a significant portion of the dataset underscores the potential value of our automated annotation system in standardizing and completing medical image metadata. This complexity mirrors the conditions in clinical settings, making our dataset particularly suitable for developing robust and generalizable deep-learning models for medical image annotation. Additionally, it improves image pseudonymization in MeDIC by detecting misclassified images, such as bone density measurement reports with patient information incorrectly labeled as X-rays.

The model identifies and annotates clear and distinct anatomical structures. For instance, the precision in detecting the boundaries in CAM of skeletal features, like the femur and humerus, is remarkably high, showcasing the model’s capability to handle well-defined body parts effectively. However, challenges remain in annotating soft tissues, overlapping anatomical features, and implants. The model’s performance slightly diminishes with low-contrast images, such as in the “Abdomen” class. These limitations suggest that further refinement is necessary for more complex scenarios.

Regarding model architecture, the pre-trained ResNet50 from our prior work achieved the best accuracy. CapsNet has low accuracy for several reasons, including insufficient data and poor initialization. The margin loss used in CapsNets might not be well-suited for all tasks. Solutions can include implementing experiments with different loss functions and adding auxiliary losses like reconstruction loss if necessary.

Regarding preprocessing and augmentation, no noticeable improvements were shown after training the data set with the proposed algorithms. However, augmenting tail classes with SMOTE slightly increased the values of Precision, F1, and Recall, indicating higher precision in tail classes. Further research should be directed at finding the balance of augmenting synthetic images.

### Limitation and future work

However, it is essential to acknowledge certain limitations and areas for future research.

While based on a dataset from patients obtaining Board Consent, further validation across diverse populations and imaging conditions is crucial to ensure generalizability. Ongoing advancements in DL and incorporating more comprehensive datasets could enhance the performance and robustness of the models.

Another limitation of this study is that the annotation was performed by a single radiologist, which introduces the potential for annotation bias. Since the examined parts involve more subjective clinical judgment, this subjectivity can reflect on the better performance of the model in Experiment II (visible parts) compared to Experiment I (examined parts). However, due to hospital workload and project interval constraints, involving multiple radiologists for comprehensive annotation was not feasible. Future work will focus on establishing more reliability by incorporating multi-expert annotations to strengthen label consistency.

Additionally, technical challenges for preprocessing include handling inverted X-ray images and determining appropriate thresholds, especially for chest and abdominal images. The model can be sensitive to lighting conditions and noise, and choosing suitable thresholds for images with varying background intensities remains challenging. In the paper, we handled the problem by augmenting the inverted image with a probability of 0.5. Extension work can be done in the context.

In terms of classification models, ResNet has long been used in medical image classification and retrieval, but has lately been outperformed by modern architectures. DenseNet improves feature reuse, EfficientNet achieves higher accuracy through compound scaling, and Vision Transformers (ViT, Swin Transformer) capture global context critical for detecting subtle lesions. These architectures generally surpass ResNet in both classification and retrieval tasks  [32]. In our previous work, we compared ResNet-50 and ViT, and the performance differences were not significant  [2]. This outcome suggests that model choice alone may not be the dominant factor when training on limited or imperfectly labeled data. Consequently, this study focused on improving the quality of the training data in close collaboration with radiologists, with the aim of providing more reliable supervision for model learning. With the resulting expert-annotated dataset, future work can systematically benchmark more advanced architectures and better assess their potential benefits for medical image classification and retrieval.

Compared to Vision-Language Models (VLMs), our proposed method with image classification allows image-only input. The model takes a medical image as input and produces a prediction from a fixed set of predefined terminologies, which is more aligned with standard clinical workflows  [33]. This design allows us to standardize model behavior to the international healthcare standard before integrating any downstream text-retrieval or reporting mechanisms. In contrast, VLMs such as BioViL or GLoRIA accept image–text pairs as inputs and typically produce embeddings, similarity scores, or generated text outputs  [34]. While this enables cross-modal tasks such as content-based retrieval or zero-shot classification, the results are not always directly interpretable or explainable for clinical decision-making  [2].

CAMs generate a heatmap overlay on the original image, highlighting the areas most influential for each predicted label and providing insight into the alignment of its decision-making process with human intuition. To further strengthen this validation, future work could involve comparing CAMs with human expert attention patterns, for instance by applying eye-tracking during expert annotation. Karargyris et al.  [35] used gaze data to inform CAM-based models during training has shown both improved accuracy and more interpretable activation maps in evaluation for chest X-ray classification tasks.

Automatic annotation with DL techniques can be applied to other imaging modalities, like MRI and ultrasound, and could boost performance real-time streamlining quality within MeDIC. Future work should focus on improving accuracy through unsupervised and online learning techniques. With the current accuracy at 0.889, there is significant room for improvement. Combining text & content-based method can be the potential solution  [36].

The proposed standardized annotation models and pipeline offer significant potential for widespread scalability across multiple data integration centers. This is primarily due to its reliance on accessible models and open-source components, which aligns with current best practices in data science  [37], offering high accessibility and low financial barriers.

## Conclusion

DICOM images from the PACS system miss or contain non-standardized and incorrect annotations. Consequently, researchers cannot collect their target dataset effectively, making image retrieval challenging.

Our study demonstrates the significant benefits and managerial impact of using Deep Learning classifiers for automated standardization annotation of X-ray images. By enhancing the accuracy, efficiency, and consistency of radiographic analysis, this approach has the potential to advance radiology research and contribute to improved healthcare outcomes. Future research should continue to refine these applications, ensuring their accuracy and practicality across various clinical use cases.

## Data Availability

The data that support the findings of this study are available from the P2N Portal https://portal.popgen.de/ but restrictions apply to the availability of these data, which were used under license for the current study, and so are not publicly available. Data are however available from the authors upon reasonable request and with permission of Ethics Committee of the Faculty of Medicine at Kiel University. The annotation pipeline and models of previous work are publicly available https://github.com/KYCheng-Ahoi/IMBODY.git.
